# Factorial Structure and Measurement Invariance of the Acceptance and Action Questionnaire-Stigma (AAQ-S) in Spain

**DOI:** 10.3390/ijerph17020658

**Published:** 2020-01-20

**Authors:** Rubén Trigueros, Noelia Navarro-Gómez, José M. Aguilar-Parra, Adolfo J. Cangas

**Affiliations:** 1 Department of Language and Education, University of Antonio de Nebrija, 28015 Madrid, Spain; rtrigueros@nebrija.es; 2 Hum-760 Research Team, Department of Psychology, Health Research Centre, University of Almería, 04120 Almería, Spain; ajcangas@ual.es; 3 Hum-878 Research Team, Department of Psychology, Health Research Centre, University of Almería, 04120 Almería, Spain

**Keywords:** psychological flexibility, experiential avoidance, stigma, prejudice

## Abstract

The objective of the present study was to validate and adapt the Acceptance and Action Questionnaire-Stigma (AAQ-S) to the Spanish context. Method: The study included the participation of 1212 subjects, with an average age of 17.12 years old. Results: The confirmatory factorial analysis revealed a number of adequate fit indices for the new version of the scale χ^2^/df = 3.24; Comparative Fit Index = 0.96; Incremental Fit Index = 0.96; Root Mean Square Error of Approximation = 0.060; Standardized Root Mean Square Residual = 0.035, in which the factorial structures displayed gender invariance. The two factors comprise the scale both exhibited high internal consistency (+0.90) and temporal stability. Conclusion: The Spanish version of the AAQ-S proved to be a robust and adequate psychometric instrument. In this sense, future lines of research focused on determining the role of psychological flexibility in stigma and the processes of change at the base of interventions could benefit substantially from the use of AAQ-S.

## 1. Introduction

In recent years, there has been increasing interest in the study of stigma and the psychological processes with the objective of advancing in the design of strategies of intervention that end with harmful consequences for those who suffer it. Stigma is a psychological process which is universal, complex and multidimensional [1,2]. It is the combination of social attitudes that affect, either directly or indirectly, the person of focus, promoting loneliness and social isolation [3,4]. Therefore, not only must mental health patients endure the symptomatology and problems derived from their conditions, they must also deal with treatment from others, which is often based on prejudices and stigmatizing beliefs. This social rejection can be observed in various aspects of life, such as access to employment, housing, and specific services, and certainly in the development of meaningful interpersonal relationships. Moreover, such exclusion also deteriorates the self-image of these individuals, further contributing to their isolation [5,6].

Studies have also shown that people who manifest a high level of stigma towards individuals with mental disorders express similar feelings towards other groups, such as ethnic minorities or those promoting sexual diversity [7]. It is therefore important to research and also bear in mind the common aspects associated with stigmatizing processes [8].

According to the Relational Frame Theory, stigma is the product of relational processes, such as the derivation and transformation of functions, which are all possible given that we are all verbal beings. From this perspective, the concept of psychological flexibility, understood as the possibility of interacting with private events that occur at the present moment, while freely choosing to stop or continue an action that provokes discomfort based on the individual’s own values. There is also evidence available in the literature that supports psychological inflexibility and experiential avoidance, such as homophobia predictor variables (e.g., [9,10]), weight self-stigma, and stigmatizing attitudes towards individuals with mental health illnesses (e.g., [11]). The clinical intervention model consistent with this theoretical framework, Acceptance and Commitment Therapy (ACT [12]), is based precisely on work to promote this psychological flexibility. To date, ACT has been more effective than other traditional interventions in the field of stigma, such as the psychoeducational protocol [13,14], these results being mediated by a statistically significant increase in their levels of psychological flexibility [15]. It is therefore important to research and also bear in mind the common aspects associated with stigmatizing processes [16]. In fact, studies have shown that people who manifest a high level of stigma towards individuals with mental disorders express similar feelings towards other groups, such as ethnic minorities or those promoting sexual diversity [7]. Thus, stigma is a general tendency to evaluate and discriminate against others based on their group membership rather than being specific to attitudes towards any one group in particular [17,18].

Despite the large number of studies that have found links between psychological inflexibility and stigma (see [16], for a review) and the evidence of psychological flexibility as an intervention method for reducing stigma towards mental disorders [15,19,20], the methods developed for evaluation purposes are relatively recent. In a study by Lillis and Hayes [20], a set of items was used to evaluate different aspects of psychological flexibility, yet it did not constitute a standardized scale. As for The Stigmatizing Attitudes Believability Scale [15], it was specifically designed to address stigma related to addiction problems, making it unsuitable for general application to measure stigma in other groups. However, there are general measures of psychological flexibility, such as the first and second versions of the Acceptance and Action Questionnaire (AAQ and AAQ-II, respectively) and its Spanish version [15,21,22]. By using these methods, the study found that the specific measures of dominance of psychological flexibility are more sensitive and applicable when research focuses on a specific problem and only one field [23,24,25].

In light of this context, Levin, Lillis, Luoma, Hayes and Vilardaga [26] set out to develop a specific measure of psychological flexibility and stigma towards mental disorder—Acceptance and Action Questionnaire-Stigma (AAQ-S). The AAQ-S was designed based on a collection of 43 items divided into 3 factors which evaluated different aspects related to stigmatizing thoughts and psychological flexibility, such as consciousness of stigmatizing thoughts, cognitive fusion with these thoughts, the distinction between the individual who has the thoughts and the thoughts themselves (i.e., self and context vs. self and content), as well as the identification of stigmatizing thoughts as barriers against carrying out valuable actions. These items were created by adapting them to already existing measures, including the AAQ [15], AAQ-II [21] and some of the general items by Lillis and Hayes [20].

In addition, the authors of the questionnaire included new items, all of which were related to general evaluations, judgments and prejudices towards others, to ensure that the measures could be utilized to assess stigma across a wide array of groups. For the final selection of the items that comprised the definitive version, five experts were called on to judge the quality of the items, with the goal of guaranteeing the maximum validity of the construct possible. Ultimately, 37 items were chosen. Following an exploratory factorial analysis, a total of nine items were eliminated due to the fact that their standardized weights in relation to their own factor were less than 0.40. Subsequently, after analyzing the validity of the construct, it was observed that one of the factors failed in the correlation and even correlated inversely with respect to the other factors, completely contrarily to what the theory establishes. Thus, the final measure was comprised of 21 items distributed on two differentiated subscales: psychological flexibility and inflexibility with stigmatizing thoughts. Additionally, it was observed that these scales displayed good internal consistency and correlated with other measures of psychological inflexibility and stigma (construct validity). In addition, AAQ-S is more strongly correlated with stigma-related measures (i.e., the Interpersonal Reactivity Index; IRI [27]; the Bogardus Social Distance Scale, SDS [28]; or the Scale of Ethnocultural Empathy, SEE; [29]).

The objective of the present study was to adapt and validate the AAQ-S of Levin et al. [26] to adolescence in the Spanish context. It was decided to work with the adolescent population for many reasons: firstly, because of the high prevalence rate of mental disorders in young people (it is estimated that in the last year, two million young people have suffered some kind of psychopathology [30]) which often carry stigma as a collateral problem that delays the search for help [31,32]; Secondly, because the instruments for measuring stigma in the adult population do not show appropriate psychometric properties for recommending their use in the adolescent population [33], and thirdly, because of the enormous number of studies that demand the need for intervention since young people are found to have important stigmatizing beliefs towards those who suffer mental health problems, such as being dangerous, unpredictable or incapable of assuming job responsibilities [34,35].

Thus, in order to provide the international community with an effective, valid and reliable tool, an exploratory factor analysis and a confirmatory factor analysis were carried out to test and confirm the factor structure of the scale. Subsequently, the reliability of the instrument was analysed using Cronbach alpha. Likewise, an invariance analysis was performed in order to test if the factorial structure of the questionnaire is understood in a similar way regardless of gender and sex, and a temporal stability analysis was also performed to determine whether the questionnaire is understood in a similar way by the same population despite the passage of time. Therefore, the hypothesis of the present study is that the confirmatory factorial analysis (CFA) of the proposed tool (AAQS-S) would offer adequate fit indices for a model with two correlated factors and said model would exhibit gender invariance and display adequate temporal stability.

## 2. Method

### 2.1. Participants

The participants in this study were 1212 teenagers (646 men and 566 women). They were between the ages of 15 and 19 (M = 17.12; SD = 1.30). This sample was used for a confirmatory factorial analysis and belonged to three randomly selected youth associations and educational institutions from 10th grade to 12th grade in the Autonomous Region of Andalusia. 

For the exploratory factorial analysis, an independent sample of 304 adolescents took part (154 male and 150 female), who were between the ages of 15 and 17 (M = 15.92; SD = 0.68). For the temporal stability analysis, an independent sample of 64 adolescents took part (34 men and 30 women), who were between the ages of 15 and 17 (M = 15.73; SD = 0.72).

### 2.2. Measures

The Acceptance and Action Questionnaire-Stigma (AAQ-S; [26]). For the purpose of measuring these factors, the Acceptance and Action Questionnaire-Stigma was validated and adapted. This questionnaire is comprised of 21 items divided between two factors: psychological flexibility (11 items) and psychological inflexibility (10 items which use reverse scoring). The subjects had to indicate their response according to a Likert scale from 1 (“Never true”) to 7 (“Always true”). It should be noted that the elements on the subscale of psychological inflexibility were classified using reverse scoring, meaning that the low scores on this subscale indicate high flexibility with stigmatizing thoughts.

### 2.3. Procedure

The back-translation method [36] was chosen to adapt and validate the Acceptance and Action Questionnaire-Stigma. This strategy consists of two steps. Firstly, a group of translators with more than 10 years of experience and training in psychology carried out a direct translation of the questionnaire to Spanish. Next, a group of translators translated the items proposed in the first translation back to their original language. The degree to which they coincided with the original version was judged according to the goodness of fit [37]. Once the final Spanish version was obtained, a group of four psychologists with more than 10 years of research experience assessed the items proposed to determine whether the items were valid for teenagers.

Having obtained the definitive questionnaire, various high school and associations in the Autonomous Region of Andalusia were contacted. They were also informed of the goal of the research and asked to offer their collaboration. As the subjects were minors, they were required to obtain parental authorization to participate in the study. Prior to being administered, the scale was tested on a small group of people to ensure that all the items were understood correctly. The scale was applied with the insistence that responses would be anonymous and that there were no true or false answers, and that participants were only asked to respond honestly. The estimated time provided to complete the questionnaire was about 10 min.

This study was carried out in accordance with the recommendations of the American Psychology Association. The entire process was conducted in accordance with the Declaration of Helsinki. All the participants also gave oral informed consent. Ethics approval was obtained from the Research Ethics Committee of the University of Almeria, Spain (Ref. UALBIO 2019/014).

### 2.4. Data Analysis

In order to determine the validity and reliability of the Acceptance and Action Questionnaire-Stigma scale, their own psychometric properties were analyzed. Firstly, a descriptive statistical analysis and heterotrait-monotrait (HTMT), to assess the validity discriminant between the two factors, the score having to be less than 0.85 [38], were conducted; furthermore, the reliability of the tool was tested by means of an internal consistency analysis (Cronbach’s alpha) and a temporal stability analysis (intraclass correlation coefficient, ICC) in order to provide evidence of the stability of the content of reports across time. Secondly, an exploratory factorial analysis (EFA) was carried out, followed by a confirmatory factorial analysis (CFA), for the purpose of testing the factorial structure of both the two-factor model and the higher-order model. Finally, a multigroup analysis was performed to examine any gender and age invariance present in the models. The statistical software used for data analysis was SPSS 24.0 (IBM, Armonk, NY, USA) and AMOS 19.0 (IBM, Armonk, NY, USA).

The sample was shown not to have a normal multivariate distribution since the Mardia coefficient proved high (86.56), the method opted for was the maximum likelihood estimation, in conjunction with a bootstrapping procedure for the CFA [39]. The estimators were not affected and can therefore be considered robust despite lack of normality [40]. With the aim of accepting or rejecting the tested model, a set of fit indices were taken into consideration: χ^2^/df, CFI (Comparative Fit Index), IFI (Incremental Fit Index), TLI (Tucker Lewis Index), NFI (Normed Fit Index), PNFI (Parsimonious Normed Fit Index), RMSEA (Root Mean Square Error of Approximation) and its 90% confidence interval (CI), and SRMR (Standardized Root Mean Square Residual). Given that χ^2^ is highly sensitive to the sample size [41], χ^2^/df was utilized and any values below 5 were considered acceptable [42]. The incremental indices (CFI, TLI, NFI and IFI) reveal a good fit with values equal to or greater than 0.95 [43], while the error indices (RMSEA and SRMR) are considered acceptable with a value equal to or less than 0.08 [44]. Finally, the PNFI reveals a good fit with values equal to or greater than 0.70.

## 3. Results

### 3.1. Analysis of Discriminant Validity, Bivariate Correlations, Descriptive Statistics and Reliability

The proportion of HTMT in the correlations between latent factors (Table 1) was 0.49, suggesting the existence of discriminant validity. Moreover, Table 1 shows the existing positive correlation between both factors, demonstrating the clear reciprocity between both factors. It must be noted that the elements on the subscale of psychological inflexibility were classified using reverse scoring, so the highest scores indicate more flexibility with stigmatizing thoughts. In addition, the average score was higher for psychological inflexibility than psychological flexibility.

With the aim of obtaining evidence of the scale’s reliability, an internal consistency analysis was conducted using Cronbach’s alpha test. The scores proved satisfactory, with 0.97 for psychological flexibility and 0.95 for psychological inflexibility.

### 3.2. Exploratory Factorial Analysis

Table 2 displays the correlations between each item and the total score of the scale, which was within a general range between 0.70 and 0.83. These results support maintaining all the items, considering the item-test correlation is higher at the cut-off point established at 0.30 [45]. The total Cronbach’s alpha was 0.82. Furthermore, the exploratory factorial analysis proved the existence of two factors, revealing a saturation factor that ranges between 0.71 and 0.87 for psychological flexibility and between 0.72 and 0.84 for psychological inflexibility.

### 3.3. Confirmatory Factorial Analysis

**Figure 1:**
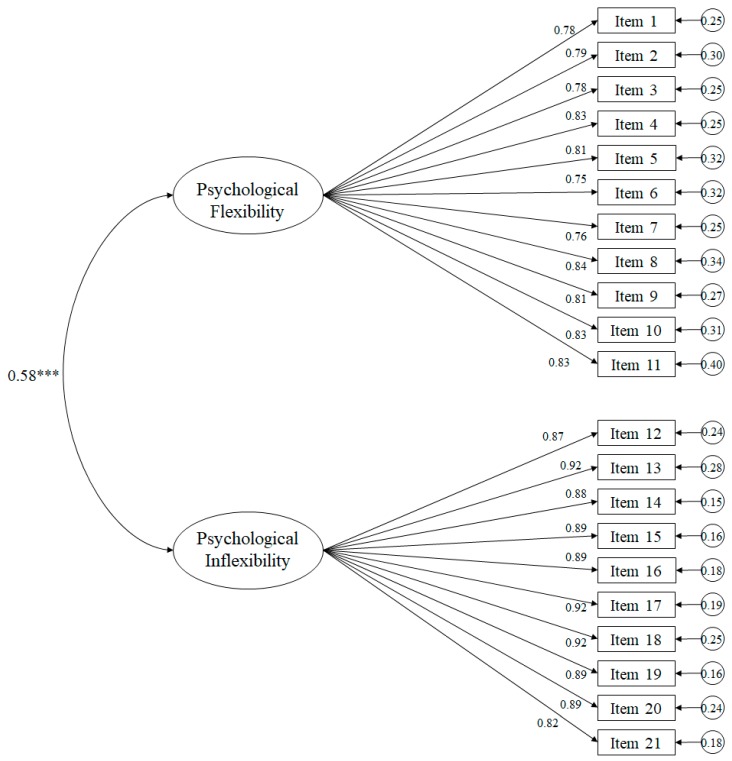
Confirmatory factor analysis of the Acceptance and Action—Stigma Scale. The ellipses represent the factors and the rectangles represent the specific items. Residual variances are presented in the small circles. Note: *** *p* < 0.001.

The fit indices of the tested model (Figure 1) proved to be suitable: χ^2^ (188. N = 1212) = 797.18, *p* < 0.001; χ^2^/df = 3.24; CFI = 0.96; IFI = 0.96; TLI = 0.96; NFI = 0.95; PNFI = 0.85; RMSEA = 0.060 (IC 90% = 0.056–0.064); SRMR = 0.035. The standardized regression weights ranged between 0.73 and 0.90, making them statistically significant (*p* < 0.001). The correlation between the factors was also statistically significant, with a figure of 0.58 (*p* < 0.001).

With regard to the higher-order model, the fit indices proved suitable: χ^2^ (192. N = 1212) = 785.34, *p* < 0.001; χ^2^/df = 2.19; CFI = 0.97; IFI = 0.97; TLI = 0.96; NFI = 0.96; PNFI = 0.88; RMSEA = 0.054 (IC 90% = 0.048–0.062); SRMR = 0.032. A relationship was revealed to exist between the higher-order factor (called acceptance and action) and both psychological flexibility (0.56) and psychological inflexibility (0.47).

### 3.4. Gender Invariance Analysis

A multigroup analysis was conducted to verify whether the factorial structure of the model exhibited gender and age invariance. Precisely as shown in Table 3 and Table 4, no significant differences were found between Model 1 (model of unconstrained) and Models 2 (model of measurement weight) and 3 (model of structural covariances). However, the results did reveal significant differences between Model 1 and 4 (model of measurement residuals).

Factorial invariance tests were performed sequentially and hierarchically. First, the configural invariance (Model 1) was established, consisting of analyzing the level of adjustment achieved by imposing only the same factorial structure. This level of adjustment would indicate to what extent the dimensional model, in its configuration, is stable or invariant in both groups, as well as playing the role of baseline of the adjustment. We then proceeded to impose new restrictions on the model. Secondly, it was imposed that the estimated factorial weights be identical for men and women constituting a test of the metrical or factorial invariance (Model 2). Thirdly, to the above restrictions it was added that the variances and covariances of the factors have identical values in their estimation for men and women (Model 3). Fourth, the equality of the error variances was attributed to analyze the reliability or invariance of the items (Model 4).

The absence of significant differences between Models 1 and 2 constitutes a minimum criterion for accepting that the structure of the model exhibits gender invariance [46].

As for the higher-order model, no significant differences were found between Model 1 (model of unconstraints), Model 2 (model of measurement weights) and Model 3 (model of structural weights). The results showed significant differences between Models 1 and 4 (model of structural covariances), Models 5 (model of structural residuals) and 6 (model of measurement residuals). These results also support the presence of gender and age invariance in the higher-order model.

### 3.5. Temporal Stability Analysis

As for the temporal stability analysis, the intraclass correlation coefficients (CCI) were calculated, along with their confidence intervals (CI), providing a score of 0.87 (CI = 0.85–0.91) for psychological flexibility and 0.88 (CI = 0.84–0.90) for psychological inflexibility.

Regarding the higher-order factor called acceptance and action, it obtained a score of 0.96.

## 4. Discussion

The objective of the present study was to validate and adapt the AAQ-S of Levin, et al. [26] to Spanish (see, Appendix A) by following a translation and adaptation process in order to ultimately analyze its psychometric properties in a study on teenagers. The analysis of these psychometric properties made it possible to confirm that it is indeed an instrument that reveals evidence of validity and reliability for obtaining a measurement of psychological flexibility in relation to stigma towards mental disorders, displaying positive correlations between the various factors that comprise the questionnaire. This instrument would be of great use to future studies dealing with assessment and intervention, and, in any case, would be more predictive than the existing general measurement of psychological flexibility (AAQ and AAQ-II).

The results of the AFC revealed rather adequate fit indices, providing positive correlations between the two factors that comprise the scale. In fact, psychological flexibility and inflexibility subscales refer to different aspects of a single construct but they do not necessarily represent distinct poles on a single dimension. For example, low psychological inflexibility does not always indicate high psychological flexibility and vice versa. These results are coherent with other similar studies (i.e., [26]). On the other hand, the results of the internal consistency test showed Cronbach’s alpha values which were greater than 0.85 on each of the two subscales (0.97 and 0.95 for psychological flexibility and inflexibility, respectively). As for the higher-order model, the values obtained were greater than 0.95. Thus, these data are consistent with the research of Wagnild and Young [47] and Vigário et al. [48].

Regarding temporal stability, acceptable values were obtained which were over 0.80 [49] on each of the two subscales and in the higher-order model. Furthermore, the stability proved invariant with respect to gender and age, which would enable it to be utilized in the future to conduct comparisons of psychological flexibility in relation to gender and age. We believe, therefore, that these results support the robustness of the scale, with the version adapted to Spanish successfully replicating the original theoretical structure. In short, this scale will prove useful as a relatively brief tool that is also easy to administer.

Taking into account the fact that this tool has shown itself to be more sensitive than the AAQ-II, correlating more strongly with other stigma measurements (e.g., [23,25]), it could be of great relevance when quantifying the impact of interventions focused on psychological flexibility towards stigmatizing thoughts.

Although the results of the present student provide psychometric support for the Spanish version of the AAQ-S, there are also some limitations that should be taken into account. Firstly, the present sample was a convenience sample and therefore, this pattern of results should be examined with other population groups that may vary by age, geographical location and other variables, given that validation of the instrument is an ongoing process. Second, future research could attempt to determine the predictive validity of the instrument through comparison with studies using similar scales (e.g., The Stigmatizing Attitudes Believability Scale [15]), as well as attempt to identify and determine psychological and behavioural predictors of prosocial behaviour, personal well-being, etc.

## 5. Conclusions

Based on the results of the present study, it can thus be concluded that the Spanish version of the AAQ-S constitutes a measure with adequate reliability and validity properties in relation to stigmatizing thoughts. Given that this population is acquiring and settling its beliefs and is therefore permeable to change, it is highly desirable to design specific interventions aimed at these stages of the life cycle [3]. The tool provided would be of great help in assessing and quantifying the magnitude of changes following anti-stigma interventions. It therefore follows that, in view to future studies, this questionnaire constitutes an invaluable tool when attempting to clarify the role of psychological flexibility in stigma, as well as the mechanisms behind the processes of change that are supposedly in action during anti-stigma interventions.

## Figures and Tables

**Table 1:** Average, typical deviation, bivariate correlations and HTMT ratio of factors.

Factors	M	SD	Range	1	2
1. Psyhological Flexibility	4.60	1.45	1–7		0.57 ***
2. Psychological Inflexibility	4.95	1.50	1–7	0.49	

Note: The value below the diagonal line correspond to the Hetertrait-Monotrait ratio (HTMT) between factors. *** *p* < 0.001.

**Table 2:** Correlations between each item and the total score on scale.

Items	Correlation Item-Test	Cronbach’s Alpha If the Item Is Eliminated	Factor Saturation of Each Item with Its Factor
1	0.79 **	0.96	0.85 F1
2	0.83 **	0.96	0.85 F1
3	0.80 **	0.96	0.85 F1
4	0.80 **	0.96	0.86 F1
5	0.78 **	0.96	0.87 F1
6	0.80 **	0.96	0.87 F1
7	0.76 **	0.96	0.85 F1
8	0.77 **	0.96	0.84 F1
9	0.78 **	0.96	0.87 F1
10	0.77 **	0.96	0.85 F1
11	0.70 **	0.96	0.71 F1
12	0.70 **	0.96	0.78 F2
13	0.71 **	0.96	0.79 F2
14	0.71 **	0.96	0.76 F2
15	0.75 **	0.96	0.72 F2
16	0.72 **	0.96	0.82 F2
17	0.78 **	0.96	0.84 F2
18	0.72 **	0.96	0.76 F2
19	0.75 **	0.96	0.77 F2
20	0.72 **	0.96	0.81 F2
21	0.77 **	0.96	0.84 F2

Note: F1 = Psychological Flexibility; F2 = Psychological Inflexibility; ** *p* < 0.001.

**Table 3:** Gender invariance analysis.

**Two-Factor Model**									
**Models**	**χ^2^**	***df***	**χ^2^/*df***	**Δχ^2^**	**Δ*df***	**CFI**	**IFI**	**RMSEA (IC 90%)**	**SRMR**
Model 1	1058.66	376	2.82	-	-	0.95	0.95	0.055 (0.051–0.059)	0.042
Model 2	1074.23	395	2.72	15.57	19	0.95	0.95	0.053 (0.050–0.057)	0.043
Model 3	1087.75	398	2.73	27.09	22	0.95	0.95	0.053 (0.050–0.057)	0.053
Model 4	1176.84	419	2.81	116.04 ***	43	0.94	0.94	0.055 (0.050–0.058)	0.054
**Higher-Order Model**									
**Models**	**χ^2^**	***df***	**χ^2^/*df***	**Δ*χ*^2^**	**Δ*df***	**CFI**	**IFI**	**RMSEA (IC 90%)**	**SRMR**
Model 1	1058.66	376	2.82	-	-	0.95	0.95	0.055 (0.050–0.057)	0.037
Model 2	1074.23	395	2.72	32.51	12	0.95	0.95	0.053 (0.050–0.057)	0.039
Model 3	1079.58	396	2.73	48.52	17	0.95	0.95	0.053 (0.050–0.057)	0.041
Model 4	1087.75	398	2.74	52.37 **	19	0.95	0.95	0.053 (0.050–0.057)	0.043
Model 5	1174.70	419	2.81	71.34 ***	26	0.94	0.94	0.053 (0.051–0.058)	0.048
Model 6	1214.31	424	2.86	100.47 ***	40	0.94	0.94	0.055 (0.053–0.059)	0.052

Note: Comparative Fit Index (CFI); Root Mean Square Error of Approximation (RMSEA); Standardized Root Mean Square Residual (SRMR); ** *p <* 0.01; *** *p <* 0.001.

**Table 4:** Age Invariance Analysis.

**Two-Factor Model**									
**Models**	**χ^2^**	***df***	**χ^2^/*df***	**Δχ^2^**	**Δ*df***	**CFI**	**IFI**	**RMSEA (IC 90%)**	**SRMR**
Model 1	799.33	376	2.13	-	-	0.95	0.95	0.058 (0.053–0.064)	0.043
Model 2	825.29	395	2.09	25.96	19	0.95	0.95	0.057 (0.052–0.063)	0.044
Model 3	827.18	398	2.08	27.85	22	0.95	0.95	0.057 (0.052–0.062)	0.048
Model 4	874.18	419	2.08	74.85 **	43	0.94	0.94	0.057 (0.052–0.063)	0.049
**Higher-Order Model**									
**Models**	**χ^2^**	***df***	**χ^2^/*df***	**Δ*χ*^2^**	**Δ*df***	**CFI**	**IFI**	**RMSEA (IC 90%)**	**SRMR**
Model 1	799.33	376	2.13	-	-	0.95	0.95	0.058 (0.053–0.064)	0.039
Model 2	825.29	395	2.09	24.87	12	0.95	0.95	0.057 (0.052–0.063)	0.039
Model 3	826.47	396	2.09	25.81	17	0.95	0.95	0.057 (0.050–0.057)	0.041
Model 4	827.18	398	2.08	29.23 *	19	0.95	0.95	0.056 (0.053–0.062)	0.043
Model 5	874.18	419	2.08	55.68 **	26	0.94	0.94	0.056 (0.053–0.063)	0.044
Model 6	891.32	424	2.10	73.98 ***	40	0.94	0.94	0.055 (0.053–0.059)	0.048

Note: Comparative Fit Index (CFI); Root Mean Square Error of Approximation (RMSEA); Standardized Root Mean Square Residual (SRMR); * *p* < 0.05; ** *p* < 0.01; *** *p* < 0.001.

## Appendix A

**Table A1:** This questionnaire was validated in Spanish.

1	Mis presuposiciones y prejuicios afectan a la forma en que interactúo con personas de diferentes orígenes.
2	Necesito reducir mis pensamientos negativos sobre los demás para poder tener buenas interacciones sociales.
3	Dejo de hacer cosas que son importantes para mí cuando se trata de alguien que no me gusta.
4	Tengo problemas para dejar de juzgar a los demás.
5	Siento que mis pensamientos prejuiciosos son una barrera significativa para ser culturalmente respetuoso.
6	Tengo problemas para no actuar en mis pensamientos negativos sobre los demás.
7	Cuando tengo pensamientos negativos sobre otros, me alejo de las personas.
8	Cuando tengo juicios sobre los demás, son muy intensos.
9	Cuando hablo con alguien creo que debo actuar de acuerdo a lo que siento por él/ella, aunque sea negativo.
10	A menudo me quedo atrapado en mis evaluaciones de lo que los demás están haciendo mal.
11	Las cosas malas que pienso de los demás deben ser verdaderas.
12	Siento que soy consciente de mis propios prejuicios.
13	Mis pensamientos negativos sobre los demás nunca son un problema en mi vida.
14	Rara vez me preocupo por tener bajo control mis evaluaciones sobre los demás.
15	Soy bueno para darme cuenta cuando tengo un prejuicio sobre otra persona
16	Cuando evalúo a alguien negativamente, soy capaz de reconocer que esto es sólo una reacción, no un hecho objetivo.
17	Soy consciente de que los prejuicios sobre los demás proceden de mi mente.
18	Está bien tener amigos sobre los que tengo pensamientos negativos de vez en cuando.
19	No me cuesta controlar mis evaluaciones sobre los demás.
20	Cuando hablo con alguien que no me gusta, soy consciente de mis evaluaciones sobre ellos.
21	Acepto que a veces tendré pensamientos desagradables sobre otras personas
